# Four Weeks of Incline Water Treadmill Exercise Can Contribute to Increase Epaxial Muscle Profile in Horses

**DOI:** 10.1155/2023/9090406

**Published:** 2023-11-07

**Authors:** Natalie Fair, Scott Blake, Roberta Blake

**Affiliations:** Writtle University College, Chelmsford CM1 3RR, UK

## Abstract

**Background:**

Water treadmill (WT) exercise is a popular modality for the training and rehabilitation of horses. However, evidence-based literature regarding the use of WT exercise, particularly using inclines, is lacking.

**Objectives:**

The aim of this study was to assess the effect of recurring inclined WT sessions on equine epaxial muscle development.

**Methods:**

Six horses completed 24 sessions of 15 minutes of WT activity over four weeks. Horses walked with water at the midcannon level at a treadmill incline of 4%. Back traces were measured at three and seven centimetres ventral to the dorsal midline at T5, T9, T14, and T18, prior to the first session (W0) and weekly for 4 weeks (W1–4).

**Results:**

Overall, the back traces demonstrated progressive increases in muscle development (*p* < 0.05), starting at W2 up to W4. At three centimetres ventral to the dorsal midline, the most to least significant increases in gross muscle development were at T18, T5, T9, and T14, respectively, and when measured at seven centimetres ventrally, the most to least significant increases were demonstrated at T5, T18, and T14. It was noted that increases in thoracic back profile musculature were mainly observed within two to four weeks of the WT intervention.

**Conclusions:**

It has been concluded that repeated WT exercise on an inclined setting has a significant effect on the rate and size of growth of equine thoracic back profile musculature. Muscle hypertrophy due to resistance training in the WT starts at 2 weeks within the programme, and it progresses as exercise continues to be performed.

## 1. Introduction

Water treadmill (WT) is a popular exercise for training and rehabilitation of horses [1]. Most of the research studies on equine WT are focused on the effects of the exercise during its practice, not on the long-term effects that can be accumulated. It has been well established that walking on a WT at different water levels will elicit different kinematics on limbs and back [2–7]. Although WT are used for rehabilitation of limbs and back injuries [1, 8], there is currently a paucity of data regarding the long-term effect of WT exercise, particularly whether the use of water treadmill (WT) exercise on an inclined setting causes an increase in musculature over the back of horses. It is however understood that due to the unnatural movement of the treadmill belt causing the horse to become unbalanced they then chose to employ the neck and back muscles in order to re-establish their natural stride pattern. Once accustomed to the UWTM, the horse can freely engage the muscles along their back without the added weight of a rider or saddle on top; the weight of the saddle and rider combined has been previously found to have a significant negative impact on the thoracolumbar posture [8]. It has also been observed that as the water level increases from the baseline, there is a greater amount of flexion caused over the lumbar region of the back [3, 4]. Interestingly, the velocity of the treadmill belt is also said to have an influence on the flexion and extension of the spine; as the velocity of the treadmill belt increases, the horse's body recruits the main muscles known for stabilising the vertebral column (*m. longissimus dorsi* and *m. rectus abdominis*) [9]. The *m. rectus abdominis* works as an antagonist muscle to the *m. longissimus dorsi* for stabilisation in the horse, and so, with the increasing water level, this muscle works to flex the thoracolumbar spine; it could be hypothesised that this will in turn have a cumulative effect on the muscle growth over the back profile of the horse [10]. The bow and string theory can be used to help visualise the flexion and extension of the horses' back; there are three main biomechanical observations of the equine back during locomotion known as the planes of movement: pelvic flexion (PF), lateral bending (LB), and axial rotation (AR) [5]. The bow and string theory relates with the flexion and extension of the thoracolumbar spine caused by the stabilisation muscles *m. longissimus dorsi* and *m. rectus abdominis* working antagonistic to one another. The *m. longissimus dorsi's* eccentric and concentric contractions are synchronised with the opposing contractions of the *m. rectus abdominis* to maintain the centre of mass over the base of support [11] in order to counteract the destabilising forces caused by the internal visceral mass during ambulation [12, 13]. However, it is important to remember that every case is different, and an increase in thoracolumbar flexion is very much dependent on the horses' spinal conformation and range of motion (ROM) of their spinal joints [8]. Previous studies carried out on ventral spinal flexion and extension in walk and trot have shown a linear increase in the muscular electromyographic activity of both the *m. longissimus dorsi* and *m. rectus abdominis* when changed from an incline of 0% to 6% [13, 14]. It could therefore be postulated that the recruitment of the stabilisation muscles during walking on an UWTM whilst on an inclined setting should in fact show an increase in facilitation and development of muscle across the thoracolumbar back profile more than walking on a levelled surface would. Remarkably, after just 10 days, UWTM training was shown to affect equine back ROM [5] There are, however, little to no studies which have been carried out that describe or, in fact, assess the effect that UWTM training has on the equine back profile musculature; further research studies such as the current study are needed in order to provide an insight into this gap in knowledge. Therefore, the aim of this study was to assess the effect of recurring inclined WT sessions on equine epaxial muscles' development. We hypothesised that the inclined WT therapy would increase muscle mass in the epaxial musculature.

## 2. Materials and Methods

The material in this manuscript has been acquired according to the guidelines set by the Animal (Scientific Procedures) Act 1986 and the Declaration of Helsinki and has been approved by the Animal Welfare and Ethics Committee of Writtle University College. The approval number is 98360534/2019. A written informed consent was obtained from the owners of the participants of the study. Veterinary surgeon consent was obtained.

### 2.1. Horses

This study involved six healthy, clinically sound, and WT-habituated horses, with a mean ± standard deviation (SD) age of 9.5 ± 3.73 years old, and the mean height was 169 ± 22 cm. The six subjects encompassed four breed types as follows: Hanoverian (*n* = 1), Thoroughbred (*n* = 1), Irish Sport Horse (*n* = 3), and Dutch Warmblood (*n* = 1). All six were sourced in Northern Ireland from the Irish Equine Rehabilitation and Fitness Centre. Horses were training and competing at varying levels mainly for dressage and showjumping. Horses were stabled at the Irish Equine Rehabilitation and Fitness Centre, and each of them was fed their own standard diets for the duration of the trial. Besides the WT exercise, horses had a walk in-hand exercise, ranging from 5 to 10 minutes a day at the start of the trial, reaching 15 to 20 minutes a day by the end of the trial. To ensure validity and reliability of the research, the same experienced handler prepared, loaded, and secured each horse in the treadmill and operated the individual treadmill programmes. The sample size was calculated using the resource equation approach [15], based on published studies assessing back musculature increase as cross-section area with different therapies, and adequate statistical discrimination has been achieved between 3 and 8 horses per group [16, 17]. Assuming that the methods differ in their intervention and outcomes, estimates by 10%, for Type I and II errors of 0.05 and 0.20, respectively, using the Bland–Altman test, we estimated a sample size between 5 and 11 horses would be needed (MedCalc® Statistical Software version 20.115).

### 2.2. Water Treadmill (WT) Exercise

The WT (ECB Aqua Treadmill, ECB Equine, UK) sessions were standardised for all horses and weeks and consisted of 24 sessions of 15-minute each over four weeks, with 6 sessions being undertaken weekly. The previous measurements were called W0, the measurements after the first week were named W1, and so on until the measurements after the fourth week of exercise, week 4, which were named W4. The water height, on every session, was the middle of the third metacarpal bone. The WT belt speed was determined by selecting a safe but also effective walking velocity that was comfortable for each horse, as assessed by the therapist, which was then maintained throughout the entirety of the data collection. Speed ranged from 3.5 to 3.8 km/h with a mean of 3.63 ± 0.14 km/h. Angle of incline for the WT belt was set at 4% to ensure all subjects could cope with the same inclination intensity.

### 2.3. Back Profile Dimension Measurements

**Figure 1:**
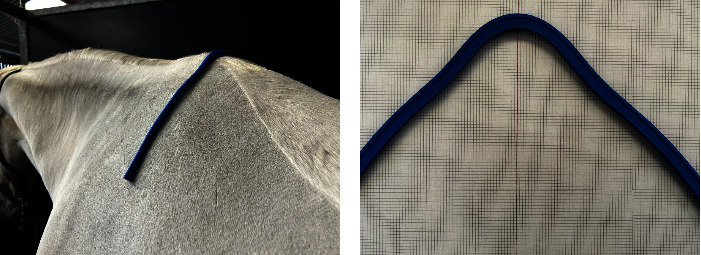
Representation of back profile dimension measurements at the T5 level. (a) The flexible ruler was used at the T5 level, over the spine, to shape the back profile. (b) The back profile was transferred to a graph paper.

Prior to the first WT treadmill session, fifth (T5), ninth (T9), fourteenth (T14), and eighteenth (T18) thoracic vertebrae were identified by palpation. Measurements were taken at each location using a saddle fitting kit (the Perfect Fit, San Francisco, USA) which includes a 50 cm flexible curve ruler (pliable metal encompassed by rubber) to shape around the dorsal surface of the back, following the contours of the body (Figure 1). A flexible curve ruler can be moulded to the contours of the spine and has been validated for assessment of transverse muscular profile [18].

**Figure 2:**
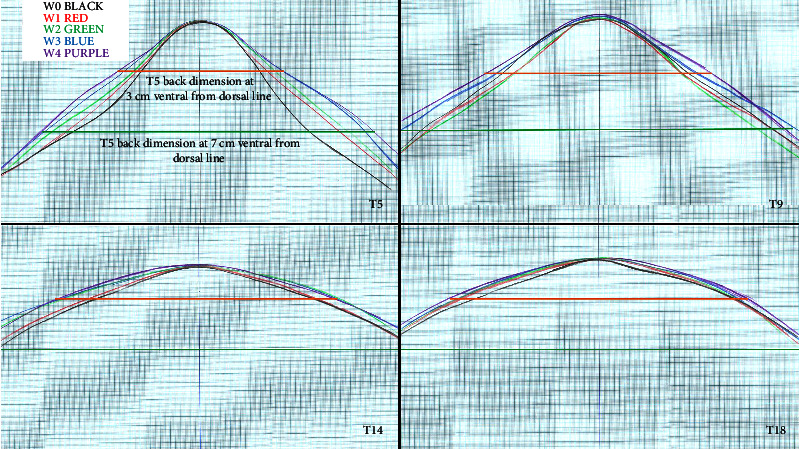
Example graphs of the changes in back dimension before the water treadmill programme (W0) and after each week of treatment (W1–W4) of water treadmill programme at fifth (T5), ninth (T9), fourteenth (T14), and eighteenth (T18) thoracic vertebrae before. The orange lines show the levels of measurement at 3 cm (orange line) and 7 cm (green line) ventral from the dorsal line.

The levels identified for the relevant vertebrae were marked with clipping a small patch of hair to ensure the measurements were repeated at the same landmarks. The subsequent outline was then mapped out on A3 graph paper (Figure 1) [18] for each timepoint on a separated sheet to denote the different measurements and avoid bias. For one single horse, besides tracing the back profiles of each timepoint on separated sheets, the profiles were also traced on a common sheet to allow the visualisation of all timepoints on single sheet, only for purposes of illustration of results. The same researcher (NF) repeated each measurement throughout the study. Before taking measurements, the assessor ensured the horse was standing squarely, with its weight evenly distributed over all four limbs, and the head and neck positions were maintained in a neutral posture with the horse's mouth level with the point of the shoulder by an assistant. All measurements were taken in a concrete flat surface adjacent to the WT. The thoracic back-profile dimension was measured immediately before the first WT session (W0) and after each 6 sessions (weekly W1–W4), totalling 5 measurements during the 4 weeks of data collection. The measurements after sessions was carried out 20 minutes after the exercises to ensure cool down, as it has been reported that immediately after exercise, the muscle cross-sectional area may change [19]. The thoracolumbar dimensions were defined as the widths, in cm, at two levels: 3 cm and 7 cm ventral to the dorsal midline (Figure 2).

### 2.4. Statistical Analysis

Once all measurements were taken, a standard ruler was used to measure the width of the back traces, in centimetres, at three and seven centimetres below the dorsal line, using each of the A3 traces collected, with data recorded in excel, and subsequently transferred to SPSS v26.0.

The mean ± SD of the back traces at each timepoint was calculated and subjected to the Shapiro–Wilk test for normality which showed parametric data. Following this, all data underwent Mauchley's test of sphericity to identify whether the assumption of sphericity had been violated, if this was the case, the Greenhouse–Geisser correction was applied to the within subjects' effects. From here, it was ascertained as to whether the back traces exhibited statistically significant increases (*p* < 0.05) between different timepoints. As all the data were parametric (*p* > 0.05) (apart from W7, M5 on T14; however, there were no outliers as assessed by inspection of the boxplots), they all underwent assessment by the means of one-way repeated measures analysis of variance (ANOVA). A post hoc analysis was then conducted with Bonferroni correction (95% confidence interval (CI)) assessments in order to determine where the statistically significant differences of the back traces lay and between which timepoints. The results report SPSS Bonferroni adjusted *p* values.

## 3. Results

An example of back-width measurements taken over the period of four weeks can be seen in a representative image of the back-profile dimensions measurement which is presented in Figure 2 (note that this is just an illustrative figure, and the measurements were taken on timepoints traced in separated sheets to avoid bias).

### 3.1. T5

**Figure 3:**
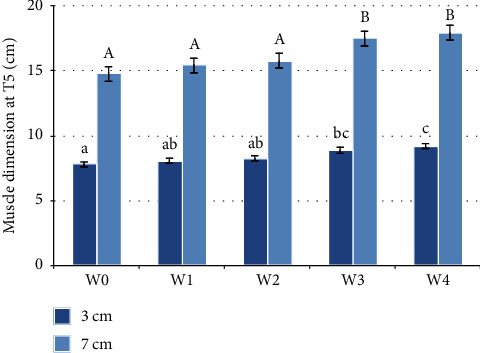
Muscle dimensions at T5 before the water treadmill programme (W0) and after each week of treatment (W1–W4) (*n* = 6). The standard error is shown. Statistically significant differences, by repeated measures ANOVA, are indicated by different letters. Lower case letters are used for measurements 3 cm ventral to the dorsal line. Capital letters are used for 7 cm ventral to the dorsal line.

The width of the T5-back traces at three centimetres below the dorsal line was statistically and significantly different at timepoints (*F*(4, 20) = 5.443, *p* = 0.004). The width of the T5-back traces at three centimetres below the dorsal line had statistically significantly increased from measurement W0 (before any WT activity) when compared with W3 (1.067 (95% CI, 0.108 to 2.025) cm, *p* = 0.035) and with W4 (1.383 (95% CI, 0.339 to 2.427) cm, *p* = 0.019). Statistically significant increases were also noted from W1 when compared with W4 (1.133 (95% CI, 0.404 to1.862) cm, *p* = 0.010) and from W2 compared to W4 (0.933 (95% CI, 0.141 to 1.726) cm, *p* = 0.029) as illustrated in Figure 3. The other pairwise comparisons were not significant (*p* > 0.05).

The width at the T5 level at seven centimetres below the dorsal line were also statistically significant at different timepoints (*F*(1.461, 7.303) = 7.271, *p* = 0.023). The test revealed a statistically significant increase from measurement W0 when compared with W3 (2.717 (95% CI, 0.125 to 5.308) cm, *p* = 0.043) and with W4 (3.150 (95% CI, 0.063 to 6.237) cm, *p* = 0.047). Statistically significant increases were also noted from W1 when compared with W3 (2.050 (95% CI, 0.661 to 3.439) cm, *p* = 0.013), and with W4 (2.483 (95% CI, 0.977 to 3.990) cm, *p* = 0.008), and from W2 when compared with W3 (1.717 (95% CI, 0.147 to 3.286) cm, *p* = 0.038), and with W4 (2.150 (95% CI, 0.537 to 3.763) cm, *p* = 0.019) as illustrated in Figure 3.

### 3.2. T9

**Figure 4:**
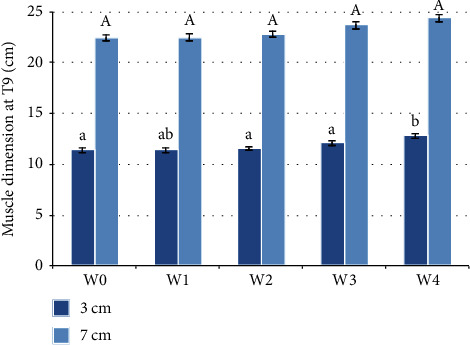
Muscle dimensions at T9 before the water treadmill programme (W0) and after each week of treatment (W1–W4) (*n* = 6). The standard error is shown. Statistically significant differences, by repeated measures ANOVA, are indicated by different letters. Lower case letters are used for measurements 3 cm ventral to the dorsal line. Capital letters are used for 7 cm ventral to the dorsal line.

The width of the T9-back traces at three centimetres below the dorsal line was statistically significant at different timepoints (*F*(4, 20) = 3.805, *p* = 0.019). Post hoc analysis with Bonferroni correction revealed that the width of the T9-back traces at three centimetres below the dorsal line had statistically significantly increased from W0 (before any WT activity) when compared with W4 (1.400 (95% CI, 0.091 to 2.709) cm, *p* = 0.040), and from W2 when compared with W4 (1.250 (95% CI, 0.136 to 2.364) cm, *p* = 0.034), and from W3 when compared with W4 (0.717 (95% CI, 0.118 to 1.316) cm, *p* = 0.028) as illustrated in Figure 4. The other pairwise comparisons were not significant (*p* > 0.05).

Traces of T9 profile at seven centimetres below the dorsal line at different timepoints were not statistically significant (*p* > 0.05) (Figure 4).

### 3.3. T14

**Figure 5:**
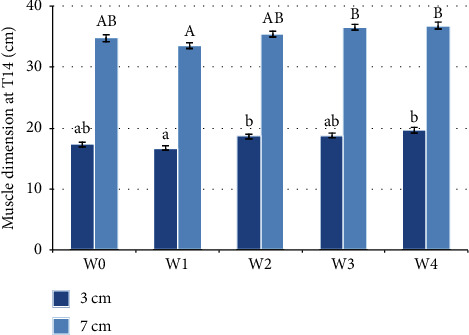
Muscle dimensions at T14 before the water treadmill programme (W0) and after each week of treatment (W1–W4) (*n* = 6). Standard error is shown. Statistically significant differences, by repeated measures ANOVA, are indicated by different letters. Lower case letters are used for measurements of 3 cm ventral to the dorsal line. Capital letters are used for 7 cm ventral to the dorsal line.

At three centimetres below the dorsal line, back traces at T14 were statistically significantly different between timepoints (*F*(4, 20) = 3.849, *p* = 0.018). The width of the T14-back traces at three centimetres below the dorsal line has statistically significantly increased from W1 when compared with W2 (1.9 (95%CI, 0.103 to 3.697) cm, *p* = 0.042) as well as when compared to W4 (2.867 (95% CI, 0.167 to 5.566) cm, *p* = 0.004), as illustrated in Figure 5.

Traces of T14 at seven centimetres were also statistically significantly different at timepoints (*F*(4, 20) = 3.108, *p* = 0.038). The width of the T14 back traces at seven centimetres below the dorsal line had statistically significantly increased from W1 when compared with W3 (2.933 (95% CI, 0.060 to 5.807) cm, *p* = 0.047) and with W4 (3.233 (95% CI, 1.037 to 5.429) cm, *p* = 0.013) as illustrated in Figure 5.

### 3.4. T18

**Figure 6:**
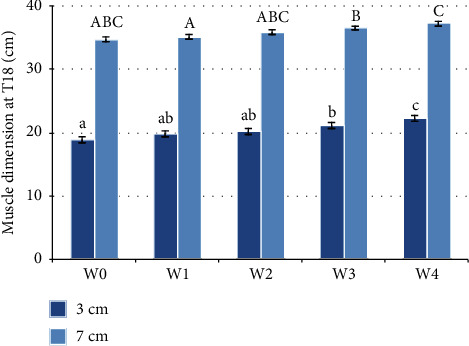
Muscle dimensions at T18 before the water treadmill programme (W0) and after each week of treatment (W1–W4) (*n* = 6). The standard error is shown. Statistically significant differences, by repeated measures ANOVA, are indicated by different letters. Lower case letters are used for measurements 3 cm ventral to the dorsal line. Capital letters are used for 7 cm ventral to the dorsal line.

Statistically significant differences at the 3 cm deep measures were observed at different timepoints (*F*(2.150, 10.751) = 5.937, *p* = 0.003). T18-back traces at three centimetres below the dorsal line have statistically significantly increased from W0 when compared with W3 (2.283 (95% CI, 0.201 to 4.366) cm, *p* = 0.037), and with W4 (3.300 (95% CI, 1.252 to 5.348) cm, *p* = 0.009). Statistically significant increases were also noted from W1 when compared with W4 (2.283 (95% CI, 1.008 to 3.559) cm, *p* = 0.006), and from W2 when compared with W4 (2.017 (95% CI, 0.320 to 3.713) cm, *p* = 0.028), and from W3 when compared with W4 (1.017 (95% CI, 0.123 to 1.911) cm, *p* = 0.033) as illustrated in Figure 6.

At seven centimetres trace widths at T18 were statistically significant at different timepoints (*F*(4, 20) = 3.551, *p* = 0.024). T18-back traces at seven centimetres below the dorsal line have statistically significantly increased from W1 when compared with W3 (1.417 (95% CI, 0.092 to 2.741) cm, *p* = 0.040), and with W4 (2.100 (95% CI, 0.813 to 3.387) cm, *p* = 0.009), and from W3 when compared with W4 (0.683 (95% CI, 0.059 to 1.307) cm, *p* = 0.037) as illustrated in Figure 6.

## 4. Discussion

The present study was conducted with the purpose of investigating the efficacy of recurring WT exercise with inclined resistance to cause increases in thoracic epaxial muscles of horses. Overall, significant increases in epaxial muscle profile have started to be shown at W3 of the WT therapy, with usually a further increase at W4. The most significant changes were seen at T5 and T18 levels, although all back points assessed have shown some increase in epaxial muscle profile. Our results, as a whole, have supported the hypothesis that WT therapy can help on the development of the epaxial muscles. To the authors' knowledge, the present study is the first of its kind to periodically measure the difference in equine back profile musculature over a four-week period, using back traces at four different points along the thoracic vertebrae. Current literature regarding the use of the WT as a rehabilitation tool is limited with respect to its efficacy in activating and building the paraspinal musculature in the thoracic region.

This study demonstrates statistically significant increases in muscle development at each of the anatomical reference points (T5, T9, T14, and T18) at three centimetres ventral to the dorsal midline, with the most significant increases in muscle development noted at T18. Although during UWT work, the thoracolumbar joint (T18/L1) sees a decrease in flexion-extension in comparison with more cranial thoracic areas [2], we could infer that deeper stabilising muscles may have been activated. It is well known that inclination of treadmill plays a role in epaxial muscle activation, particularly *longissimus dorsi* [13]. When treadmill inclination changed from 0 to 6%, EMG activity of the *longissimus dorsi* began and ended later; therefore, a longer activity duration was noted [13]. Lower inclinations such as 3% has also been reported to increase EMG values for the longissimus dorsi [20], and this effect of the incline has contributed towards the increased epaxial profile reported in our study, as we have used an incline of 4%.

The next most statistically significant increases in muscle growth were found at T5, noted as the highest point of the withers, and as such is subject to the greatest level of shear forces during ridden exercise due to direct transmission of rider's weight through the stirrups, which may affect the muscles responsible for protraction and retraction of the forelimbs [21]. It could be suggested that the muscle development in this region may be due to the horse being able to freely engage the muscles in this area without the negative influence of a rider or saddle during the WT exercise [4]. In addition, it could be postulated that the increases in size of the musculature were facilitated by the water level being set low enough to allow the horses to lower their head and neck, causing traction on the withers from the nuchal and supraspinous ligaments [22, 23]. This creates cranial thoracic flexion [24] and activates the vertebral stabilisation muscles. Furthermore, research has reported that WT exercise increases considerable activation of *m. splenius* [20], and as this muscle inserts at T5, its activations during WT exercises could have contributed towards the muscle profile observed at this level in our study.

Statistical significance was also apparent at T9 between different timepoints, which may be associated with the specific anatomical characteristics of the vertebrae involved in the adjacent articulations, which determines the degree of movement at each joint complex [25]. The body of vertebral segments T2–T9 is shorter but provides larger points of attachment for the nuchal and supraspinous ligaments as well as for the relevant muscles which produce the movements of the back [26]. Therefore, T2-T9 possess greater amounts of intervertebral movement, displacement, and axial rotation between these joint complexes, due to the elasticity of the ligaments [23, 25, 26]. The aforementioned biomechanical characteristics of the ninth thoracic vertebrae may have contributed to muscle build by allowing increased spinal ROM, stimulating antagonistic epaxial and hypaxial muscle contractions to resist excessive displacement of the vertebral column [27]. Furthermore, it has been discovered that movements of the back, limbs, head, and neck are closely associated [25, 28]; therefore, it can be assumed that exercising on an incline will require synergistic contractions of both the back and hindlimb muscles to produce propulsion in the sagittal plane [12].

We therefore hypothesise that the benefits of the incline associated with the benefits of water at midcannon bone have created a synergic benefit for the muscular development in our study.

The vertebral segment which revealed the least differences in muscle build between the various timepoints was T14, only demonstrating a statistically significant average increase of 2.9 cm between week one and four of WT intervention. Upon closer inspection of the results, apart from an initial decrease following the first week of WT exercise, it is clear that a smaller cumulative increase in gross muscle size occurred each week; however, these may have been too minimal to reach statistical significance. The statistically significant increase in muscle mass between week one and four may have occurred as the greatest amounts of axial rotation and lateral bending occur at the vertebral level of T14 [26, 29] due to the presence of asternal ribs, which are indirectly attached to the sternum [26]. Hence, the increased axial rotation and lateral bending experienced in this vertebral region, along with the increased dorsoventral flexion-extension caused by exercising on an incline [12, 14, 30], are both expected to have influenced the facilitation and therefore also the gradual increase in mass of the *m. longissimus dorsi* and *m. rectus abdominis* in the current study as they are required for vertebral stabilisation in the horse [30]. What is worthy of note is that the timing of increases correlate with the effect of resistance training and the process of hypertrophy, whereby a minimum of two to four weeks of resistance training are required in humans to facilitate skeletal muscle hypertrophy [31].

At 7 cm, our results indicated that the most significant increases in overall thoracic musculature development occurred at T5 and T18. A possible explanation for the differences noted between the back traces at three and seven centimetres could be attributable to the function of the hypaxial muscles which are situated ventrally to the transverse processes of the spine. The hypaxial muscles mainly function to produce flexion of the cranial thoracic spine, while the *m. rectus femoris* is activated to counter spinal extension [32]. Furthermore, as the *m. rectus abdominis* inserts onto the head of the femur, it could be postulated that this muscle plays a role in the synergistic contractions of the back and hindlimbs during forward propulsion [12] possibly explaining the significantly increased muscle mass in the region.

Comparatively, the next most statistically significant increases in thoracic muscle development were observed at T18. It could be inferred that the increased muscle growth observed when measured further ventrally could be linked to the slightly more lateral and ventral origin of the *m. obliquus abdominis externus* on the lateral surface of ribs 4–18 and the thoracolumbar fascia. Likewise, the *m. rectus abdominis* is recruited during the second half of the stride in the region of T18 once the forelimb is protracted and ipsilateral hindlimb is retracted to counter the excessive thoracolumbar extension caused by the downward movement of the internal visceral mass [27], possibly further explaining the increase in muscle development when exercising against increased resistance from both the water [7] and inclination [13, 14]. Statistical significance was also evident at T14, although not to the same extent as T5 and T18 as only two of the timepoints demonstrated increases in gross muscle development. This could also be attributable to the more controlled exercise in the sagittal plane without the added weight of a saddle or rider [4, 33] which may allow for more symmetrical muscle development than when exercising on land [14]. As for the significant increases in thoracic musculature observed at T14, the greatest amounts of axial rotation and lateral bending occur in this region during locomotion [26, 29]. Moreover, the horse also shows increases in hindlimb ROM when walking in water [7]. Research by Mooij [5] has also suggested that in order to produce hindlimb movement in water, there must first be a further increase in axial rotation of the caudal thoracic spine, which may explain the resulting increased muscle development at the level of T14.

No statistically significant increases in thoracic back-profile musculature were observed at T9 when measured at 7 cm; however, there was evidence of cumulative weekly increases in the back-trace widths when compared to the individual means.

Overall, our research agrees with a study recently presented, which performed a semiqualitative analysis of muscle development in horses undergoing WT work and has concluded that WT can increase thoracic epaxial muscle development, after 20 weeks of exercise [34]. However, our study has found earlier improvements in muscle development at thoracic epaxial musculature. We attribute this earlier muscle development to the fact that we have used an incline on the WT, while the previous study [34] did not mention incline so was probably performed under flat WT. As mentioned before, both water and incline have an influence on epaxial musculature activation and, therefore, growth. Hence, we believe that the combination of these two elements have contributed to the earlier positive results observed in our study.

That being said, our study also had its limitations. The first could be small sample size, which could lead to the assumption that our results may lack ecological value; that being said, the majority of our results demonstrated statistical significance between different timepoints, which warrants further investigation with a larger sample size in the future to more precisely evaluate the effects of the intervention, to allow more definitive conclusions to be drawn. A potential limitation concerning the repeated measures study design is the effect that the treatment intervention has on subsequent treatments, the lack of a control group and not having flat UWT and inclined dry treadmill groups to ascertain which independent variable (water or incline) was more relevant in eliciting the observed changes, or if both interventions will contribute towards muscle hypertrophy. As this study utilised a longitudinal layout over the course of four weeks, it could be said that biased estimates of the treatment efficacy may be made due to the carry-over effect of the treatment modality. However, this study design emphasised comparisons within each horse over time, and as horses had a similar trend on muscular growth, we can attribute this to the exercise on the inclined WT with a good certainty, although we cannot attribute the effects to the water or incline separated. Despite all limitations, it is our belief that the current study presents an insightful account into the rate and size of paraspinal muscle growth in the thoracic region through the implementation of repeated WT exercise with incline resistance. Another limitation is that as a result of being reshaped repeatedly, the flexible curve ruler is susceptible to deterioration over time; therefore, it is advised to frequently replace the flexible curve ruler used for data collection to ensure the results remain consistent. We have used the same piece of equipment all throughout the measurements, and we have not noticed any significant misshapen of the ruler. We also acknowledge that there are other more objective means to measure epaxial muscle changes in size as evaluation of cross-sectional area with ultrasound scans as described in the literature [16, 35]. However, our choice to use the flexible curve ruler was due to equipment availability and the fact that there is a validation for the use of this tool^9^.

## 5. Conclusions

The results of this study support the original hypothesis, revealing that repeated WT exercise on an inclined setting does in fact have a significant effect on the rate and size of growth of the equine thoracic back profile musculature, although further research should be conducted to determine which intervention is more relevant, incline or water, and if both interventions have a synergic effect, as well as which specific muscles are recruited during WT intervention with incline resistance and to establish the increases in the cross-sectional area of the individual muscles with ultrasound scans rather than just the total increases in overall muscle development.

## Data Availability

The data that support the findings of this study are available from the corresponding author upon reasonable request.
